# Numerical Simulation Study on the Influence of Fracture on Borehole Wave Modes: Insights from Fracture Width, Filling Condition, and Acoustic Frequency

**DOI:** 10.3390/s24123955

**Published:** 2024-06-18

**Authors:** Ziran Gao, Dong Wu, Hongliang Wu, Peng Liu, Ming Cai, Chengguang Zhang, Jun Tang

**Affiliations:** 1 Key Laboratory of Oil and Gas Resources and Exploration Technology of Ministry of Education, Yangtze University, Wuhan 430100, China; 18171593372@163.com (Z.G.); zhangcg@yangtzeu.edu.cn (C.Z.); tangjun@yangtzeu.edu.cn (J.T.); 2 College of Geophysics and Petroleum Resources, Yangtze University, Wuhan 430100, China; 3 Information Center, CNPC Engineering Technology Research Institute, Beijing 102206, China; lh_wudong@cnpc.com.cn; 4 PetroChina Research Institute of Petroleum Exploration & Development, Beijing 100083, China; wuhongliang@petrochina.com.cn (H.W.); liupeng1987@petrochina.com.cn (P.L.)

**Keywords:** fractures, acoustic logging, amplitude attenuation, numerical simulation, borehole wave modes

## Abstract

Unconventional reservoirs, such as shale and tight formations, have become increasingly vital contributors to oil and gas production. In these reservoirs, fractures serve as crucial spaces for fluid migration and storage, making their precise assessment essential. Array acoustic logging stands out as a pivotal method for evaluating fractures. To investigate the impact of fracture width, fracture-filling conditions, and acoustic frequency on compressional and shear waves, a three-dimensional variable mesh finite difference program was employed for acoustic logging numerical simulation. Firstly, numerical models representing fractured formations with varying fracture widths and distinct fluid-filling conditions were established, and array acoustic logging numerical simulations were conducted at different frequencies. Subsequently, the waveform data were processed to extract acoustic characteristic parameters, such as velocities and amplitude attenuations of compressional and shear waves. Finally, a quantitative analysis was conducted to examine the variation patterns of characteristic parameters of refracted compressional and shear waves in relation to fracture properties. The research results indicate that amplitude attenuation information derived from borehole wave modes is particularly sensitive to the changes in fracture properties. As fracture width increased, we observed a significant amplitude attenuation in both compressional and shear waves, proportional to the logarithm of the attenuation coefficients. Furthermore, when the fracture width was constant, gas-filled fractures exhibited more prominent amplitude attenuation than water-filled fractures, with shear wave attenuation being more sensitive to the filling material. Moreover, from a quantitative perspective, the analysis revealed that the attenuation coefficients of refracted compressional and shear waves exhibited an exponential variation with gas saturation. Notably, once fracture width and filling conditions were established, the amplitudes of compressional and shear waves at the dominant frequency of 40 kHz were significantly reduced compared to those at 8 kHz, accompanied by increased attenuation. Subsequent quantitative analysis revealed that, when the product of fracture width and dominant frequency remains constant, the corresponding attenuation coefficient ratios approach 1. This indicates that the attenuation process of acoustic propagation in fractured media follows the principle of acoustic similarity. The findings of this study provide reference for further research on fracture property evaluation methods based on array acoustic logging data.

## 1. Introduction

With the development of oil and gas exploration, unconventional tight and fractured reservoirs have become key areas of focus in the industry. These reservoirs heavily rely on fractures as essential conduits for fluid migration and storage, and their development significantly impacts reservoir performance [1,2,3,4]. Consequently, the evaluation of fractures in these reservoirs has become critical. The research on fracture evaluation is mainly carried out from two aspects: physical experiments [5,6,7,8,9,10,11] and numerical simulations [12,13,14,15,16,17,18]. Currently, various methods are used to assess fractures, such as the dual lateral resistivity difference method, dipole shear wave anisotropy method, borehole wave modes’ attenuation method, Stoneley wave reflection coefficient method, and others. Among these methods, acoustic logging [19,20,21,22,23] stands out as a crucial tool for precise fracture assessment. Dual lateral resistivity logging, renowned for its extensive detection capability and high sensitivity to adjacent geological anomalies, is particularly suited for identifying fractures. Ge et al. conducted a physical simulation and feature analysis of near borehole fractures, revealing that dual lateral resistivity increased as fractures moved farther away from the borehole [24]. Additionally, as the angle of fractures increased, the deep lateral resistivity also increased, whereas shallow lateral resistivity remained relatively unchanged. Wu et al. proposed a three-dimensional finite element method to simulate array lateral logging and developed a physical simulation system to verify the accuracy of the three-dimensional finite element method [25]. The effects of anisotropy and inclination on the array lateral logging were then analyzed. Wang et al. studied the dual lateral resistivity logging responses in horizontal wells, dipping anisotropic formations, and fractured carbonate formations by using a three-dimensional finite element program [26]. Their findings revealed that within fractured formations, the resistivity decreased as the fracture width increased. For evaluating fractures using the dipole shear wave anisotropy, Liu et al. proposed a new method for the fast processing of cross-dipole anisotropy [27]. The method significantly improved efficiency and stability in data processing, leading to increased accuracy in fracture assessments. Zhou established a calculation model for rock mechanical parameters based on 3D anisotropy [28]. This approach enhanced the accuracy of reservoir positioning. Mehrdad et al. investigated the influence of frequency bandwidth on the assessment of dipole shear wave anisotropy [29]. Their research demonstrated that employing dipole shear wave imaging tools provides higher accuracy in assessing anisotropy in porous media. In the context of evaluating fractures through the borehole wave modes’ attenuation method, Liu et al. delved into the influence of fracture width and inclination on shear wave and Stoneley wave attenuation in the borehole environment by establishing a three-dimensional variable mesh time-domain finite difference model [30]. The study highlighted the sensitivity of Stoneley wave attenuation to fractures, although it did not consider the influence of compressional wave attenuation. Cai et al. conducted physical experiments to investigate the impact of microcracks on shear wave propagation [11]. They established quantitative relationships between shear wave attenuation and changes in microcrack width, but they did not consider the effects of fracture-filling conditions and acoustic frequency. Xu performed the finite difference method to explore full wave propagation in porous formations [31]. Their study explained clearly the relationship between horizontal fractures and borehole Stoneley waves. The results revealed that Stoneley waves formed reflections at the elastic-pore interface, accompanied by energy attenuation. Chen et al. conducted numerical simulations in a borehole surrounded by a cracked porous formation to analyze the attenuation characteristics of multipole waves in porous dense media [32]. Their study revealed that the primary cause of borehole wave modes’ attenuation was the attenuation of formation shear waves induced by fractures, providing a reference for assessing the degree of fracture development. The Stoneley wave reflection coefficient, exhibiting high sensitivity to fracture width, is frequently used for fracture identification. Cao et al. investigated the relationship between rough fracture width and the attenuation coefficient through numerical simulations and physical experiments [33]. They established a direct relationship between the Stoneley wave attenuation coefficient and fracture width. Wang et al. introduced a technique of the non-parametric spectral estimation [34]. By utilizing the finite difference method, they derived phase dispersion properties and Stoneley wave attenuation coefficients. This method enabled the accurate determination of fracture locations.

As noted earlier, previous research has primarily focused on fracture evaluation methods such as the dual lateral resistivity difference method, dipole shear wave anisotropy method, borehole wave modes’ attenuation method, and Stoneley wave reflection coefficient method. However, there are relatively few studies using P-waves and S-waves to evaluate fractures. What is more, the effects of the fracture-filling condition and acoustic frequency are hardly considered when using the borehole wave modes’ attenuation method. Therefore, numerical models representing fractured formations with varying fracture widths and distinct fluid-filling conditions are established, and array acoustic logging numerical simulations are conducted at different frequencies. This research introduces a significant innovation by considering the effects of fracture-filling conditions and acoustic frequency on acoustic propagation in fractured formations. The findings provide valuable guidance and reference for the comprehensive evaluation of fractures using array acoustic logging data. In the following sections, the numerical simulation method and model parameter setting are first described. Then, the numerical simulation program is verified. Next, the influencing factors of borehole wave modes are discussed from three aspects through numerical simulation: fracture width, fracture filling, and acoustic frequency. The quantitative relationship between characteristic parameters of wave modes and fracture properties is obtained. Finally, the conclusions are drawn according to the results obtained from this study.

## 2. Numerical Simulation Method of Acoustic Logging in Fractured Formation

### 2.1. Parameters of Numerical Model

**Figure 1:**
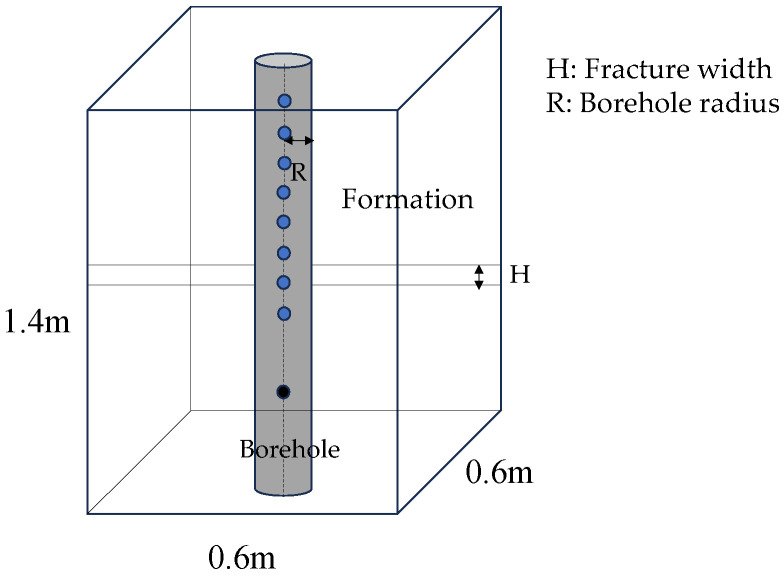
Borehole model.

**Figure 2:**
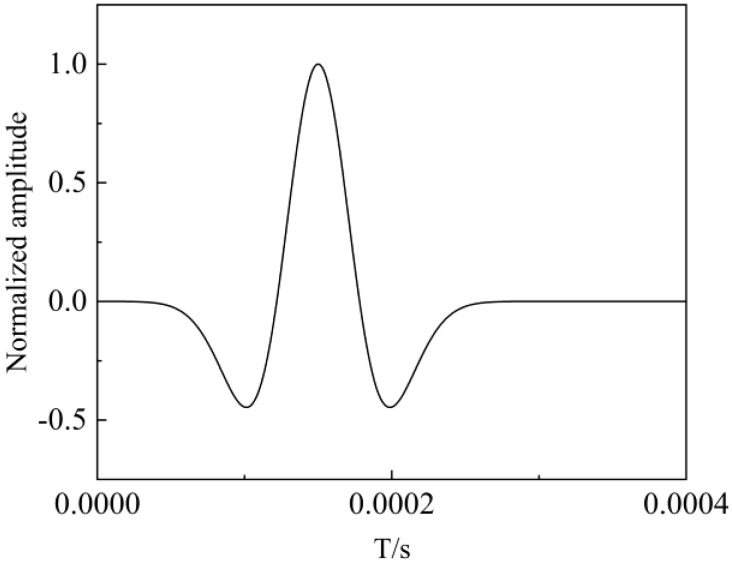
Source excitation signal.

A fractured formation model with a borehole was established by a three-dimensional variable grid finite difference program, as illustrated in Figure 1. The blue dot is the receiver and the black dot is the transmitter. The size of the model is 0.6 m × 0.6 m × 1.4 m. The borehole radius R is 0.1 m, and the fracture width is set to H. The time step is 2.887 µs, with a grid step of 2 cm. The formation outside the borehole is a uniform elastic solid formation. In the simulation, a monopole point source was employed, with the excitation signal being a deformed Ricker wavelet (as shown in Figure 2) with a central frequency of 8 kHz. The source excitation signal is expressed as Equation (1).

(1) $$f(t)=\frac{4}{3}w^{2}t^{2}e^{-\frac{w_{0}}{\sqrt{3}}}\sin(w_{0}t)$$

### 2.2. The 3D Variable Mesh Finite Difference Numerical Simulation Method

The finite difference method was employed in this study to simulate the sound field within the borehole based on the specified parameters [35,36,37]. We utilized a three-dimensional finite difference variable grid program to simulate the sound environment within the borehole. The fundamental principle of the finite difference method involves numerically calculating the properties of the medium and physical quantities using the wave equation. Subsequently, iterative processes involving velocity and stress were employed to determine the sound field at different time steps and locations. In the established three-dimensional finite difference model, small grids are necessary at the locations of fractures due to their thin nature within the formation. However, using small grids uniformly throughout the fracture regions would result in extensive computation and slow processing speeds. To address this, a non-uniform grid approach is employed, with small grids at fracture locations and larger grids elsewhere. This methodology effectively improves both the accuracy and efficiency of the calculations [38].

The formula for velocity and stress in the anisotropic elastic wave equation in Cartesian coordinates is as follows [39]:

(2) $$\rho \dot{v}=D\times T$$

(3) $$\dot{T}=C\times D^{T}\times v$$

(4) $$v^{T}=(v_{x},v_{y},v_{z})$$

(5) $$T^{T}=(T_{xx},T_{yy},T_{zz},T_{yz},T_{xz},T_{xy})$$

where ρ(x) is the fluid density of the medium, v(x,t) is the vibration velocity vector of the elastic medium, and T(x,t) is the stress vector. $\dot{v}\left(x,t\right)$ is the derivative of the velocity vector for time, and $\dot{T}\left(x,t\right)$ is the derivative of the stress vector for time. *D* and *C* are defined as follows:

(6) $$D=\left(\begin{array}{cccccc} \frac{\partial }{\partial x} & 0 & 0 & 0 & \frac{\partial }{\partial z} & \frac{\partial }{\partial y}\\ 0 & \frac{\partial }{\partial y} & 0 & \frac{\partial }{\partial z} & 0 & \frac{\partial }{\partial x}\\ 0 & 0 & \frac{\partial }{\partial z} & \frac{\partial }{\partial y} & \frac{\partial }{\partial x} & 0 \end{array}\right)$$

(7) $$C=\left(\begin{array}{cccccc} c_{11} & c_{12} & c_{13} & 0 & 0 & 0\\ c_{12} & c_{22} & c_{23} & 0 & 0 & 0\\ c_{13} & c_{23} & c_{33} & 0 & 0 & 0\\ 0 & 0 & 0 & c_{44} & 0 & 0\\ 0 & 0 & 0 & 0 & c_{55} & 0\\ 0 & 0 & 0 & 0 & 0 & c_{66} \end{array}\right)$$

**Figure 3:**
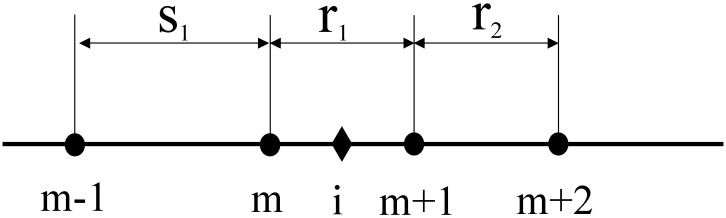
Finite difference diagram of irregular variable grid.

Figure 3 depicts the diagram of the irregularly variable grid finite difference in the Z-direction, featuring a fourth-order spatial accuracy irregular grid differencing operator.

i is the midpoint of node m and m+1, and Φ is the field quantity at node i. η [40] is the fourth-order spatial precision difference operator with grid changes, and Formula (8) characterizes the linear combination of the field quantity Φ.

(8) $$\left\{\begin{array}{l} \frac{\partial \Phi }{\partial x}=\eta_{1}\Phi_{m+1}-\eta_{2}\Phi_{m}+\eta_{3}\Phi_{m+2}-\eta_{4}\Phi_{m-1},\\ \eta_{1}=\frac{1}{4}\frac{{r_{1}}^{2}+4r_{1}r_{2}+4s_{1}s_{2}}{r_{1}r_{2}(r_{1}+s_{1})},\\ \eta_{2}=\frac{1}{4}\frac{{r_{1}}^{2}+4r_{1}s_{1}+4r_{2}s_{1}}{r_{1}s_{1}(r_{1}+r_{2})},\\ \eta_{3}=-\frac{1}{4}\frac{{r_{1}}^{2}}{r_{2}(r_{1}+r_{2})(r_{1}+r_{2}+s_{1})},\\ \eta_{4}=-\frac{1}{4}\frac{{r_{1}}^{2}}{s_{1}(r_{1}+s_{1})(r_{1}+r_{2}+s_{1})}, \end{array}\right.$$

### 2.3. Verification of Numerical Simulation Program

**Figure 4:**
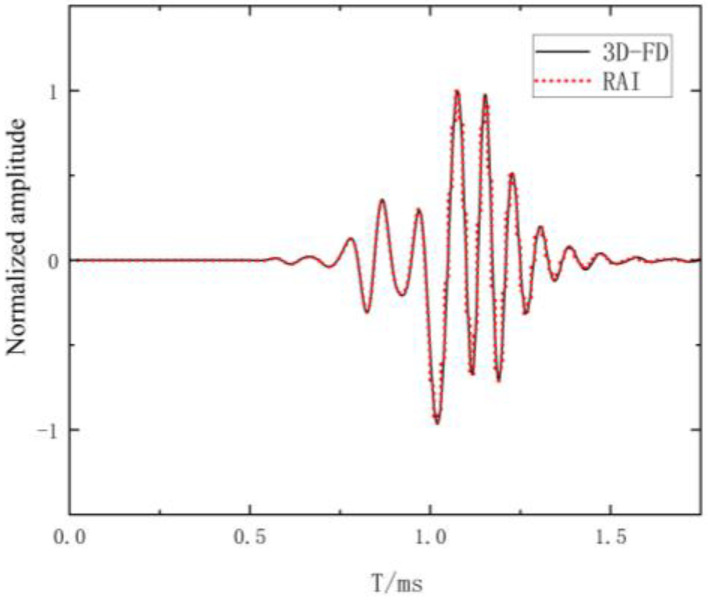
Comparison between finite difference (3D-FD) and real axis integral (RAI).

**Figure 5:**
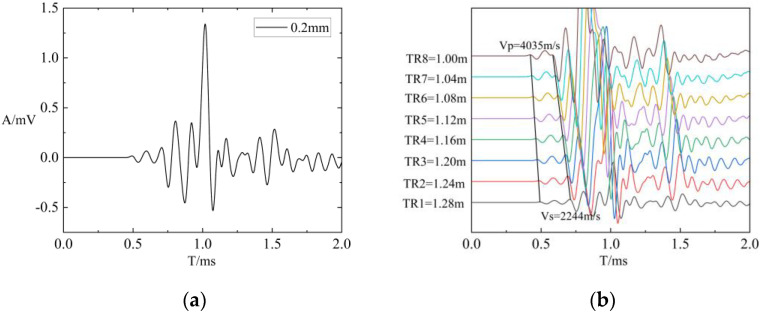
Waveform of a borehole formation model with a fracture width of 0.2 mm: (**a**) waveform with a 1.28 m TR spacing; (**b**) array waveform.

To validate the accuracy of the three-dimensional finite difference program, two verification approaches will be employed: one without fractures and one with fractures. Firstly, the waveform of the borehole pattern without fracture was calculated based on the three-dimensional finite-difference numerical simulation program and compared with the real-axis integral diagram. As shown in Figure 4, it can be observed that the primary wave modes, such as refracted compressional waves, shear waves, and Stoneley waves, exhibit consistency with the real-axis integral waveform. This validates the effectiveness of the finite difference method and program in the absence of fractures. Secondly, a three-dimensional finite difference numerical simulation program was used to compute the array waveforms of a borehole formation model with a fracture width of 0.2 mm. As illustrated in Figure 5, the results indicate that the primary wave modes, including refracted compressional waves, shear waves, and Stoneley waves, are well defined and exhibit clear characteristics, aligning with theoretical expectations. Further validation was conducted with a fracture width of 0.2 mm by comparing the refracted compressional and shear wave velocities of eight waveforms generated at different Transmitter–Receiver (TR) spacings. These velocities were calculated by using the STC (Slowness Time Coherence) method, resulting in a compressional wave velocity of 4035 m/s and a shear wave velocity of 2244 m/s. Comparing the data to the parameters of the model (detailed in Table 1) demonstrates that the calculated refracted P-wave and S-wave velocities are consistent with the P-wave and S-wave velocities in Table 1, confirming the accuracy of the finite difference method and program under fracture conditions. The verification conducted for both cases, with and without fractures, validates the accuracy of the finite difference method and program.

## 3. Numerical Simulation and Analysis of Acoustic Logging in Fractured Formation

This paper aims to investigate the impact of changes in fracture attributes on the characteristic parameters of refracted P-waves and S-waves. To this end, a three-dimensional finite difference method was employed to conduct numerical simulations of array acoustic logging in fractured formation. The research specifically explores the effects of fracture width, fracture filling, and acoustic frequency on borehole wave modes.

### 3.1. Influence of Fracture Width on Borehole Wave Modes

**Figure 6:**
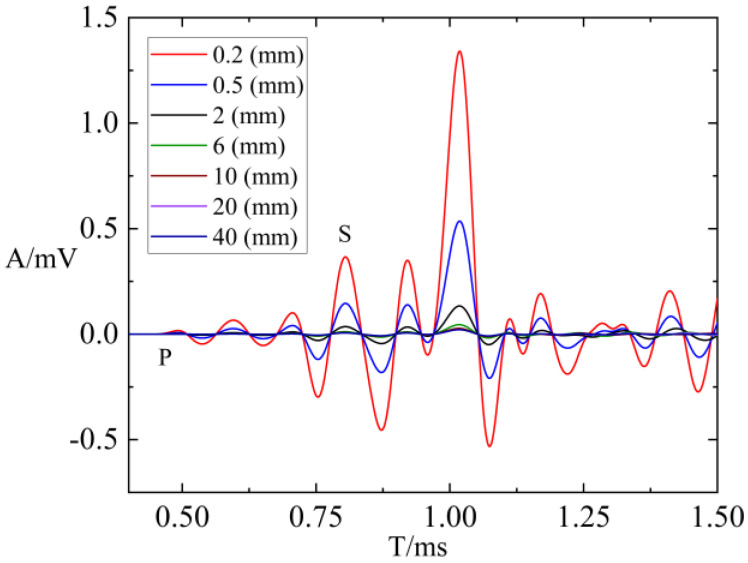
Comparison of water filling with different fracture widths.

In the simulation calculations using the finite difference program, the source dominant frequency was set to 8 kHz, with a Transmitter–Receiver spacing of 1.28 m. Both the borehole and fractures were filled with water, while other medium parameters were outlined, as shown in Table 1. To investigate the influence of fracture width on borehole wave modes, various fracture widths were defined. Figure 6 presents a waveform comparison for fracture widths of 0.2 mm, 0.5 mm, 2 mm, 6 mm, 10 mm, 20 mm, and 40 mm. As shown in Figure 6, an increase in fracture width leads to a significant decline in the waveform amplitudes of both refracted P-waves and S-waves, accompanied by a notable increase in attenuation. This observation highlights the sensitivity of these waves to changes in fracture width. However, it is worth noting that beyond a certain threshold, the amplitude variation trend in refracted P-waves and S-waves becomes less pronounced. This is due to the increasing presence of water within the fracture as its width increases, resulting in progressively greater energy attenuation in both the P-waves and S-waves. Eventually, this attenuation reaches a plateau and stabilizes. In summary, the results underscore the significant influence of fracture width on borehole wave modes. As fracture width increases, the amplitudes of refracted P-waves and S-waves diminished noticeably, and the attenuation effect became more pronounced. However, beyond a certain threshold, the amplitude changes in P-waves and S-waves became less evident. The findings of these studies are crucial for both theoretical and practical applications. They significantly contribute to the meticulous assessment of fracture property parameters and offer valuable insights into the investigation of rock mechanics parameters within geological formations.

To conduct a thorough quantitative analysis on the influence of fracture widths on borehole wave modes, the waveform data obtained under different fracture widths were thoroughly analyzed. Characteristic parameters of the wave modes were subsequently extracted and analyzed, including the calculation of the attenuation coefficient (α) for refracted P-waves and S-waves by using Equation (9) [11].

(9) $$\alpha =C\mathrm{lg}(\frac{A_{0}}{A})$$

where *C* is constant. When *C* equals 20, the unit of attenuation (α) is expressed in decibels (dB); *A*_0_ is the refracted P-wave and S-wave amplitude measured without fractures, mV; and *A* is the refracted P-wave and S-wave amplitude measured when there is a fracture, mV. By employing Equation (9), we calculated the attenuation coefficients of refracted compressional and shear waves for all considered fracture widths, establishing the relationship between fracture width and the attenuation coefficients of refracted compressional and shear waves.

**Figure 7:**
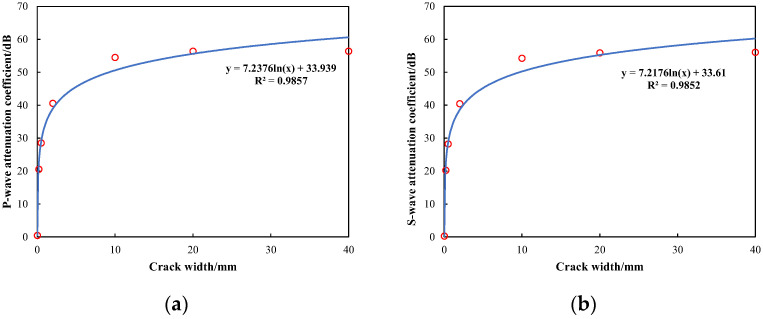
Variation in attenuation coefficient with fracture width: (**a**) relation of the attenuation coefficient of P-waves with fracture width; (**b**) relation of the attenuation coefficient of S-waves with fracture width.

Figure 7 illustrates the relationship between the attenuation coefficient of wave modes and fracture widths. Specifically, it presents the attenuation coefficients for fracture widths of 0.2 mm, 0.5 mm, 2 mm, 10 mm, 20 mm, and 40 mm. The diagram demonstrates a logarithmic relationship between the attenuation coefficients and the fracture widths. Notably, when the fracture width is within the range of 2 mm, the attenuation coefficients for refracted P-waves and S-waves exhibit a sharp increase as fracture width increases. However, as the fracture width increases to 10 mm, the attenuation coefficients for both waves tend to stabilize, with a more gradual increase in attenuation. This attenuation behavior can be attributed to the wave reflection at the fracture interface and wave energy absorption in the fracture fluid. Consequently, the rate of attenuation in micro-fractures changes rapidly, indicating that P-waves and S-waves are highly sensitive to changes in the width of micro-fractures. On the contrary, with an increase in fracture width, the attenuation rate tends to stabilize, indicating a decreased sensitivity of both waves to macro-fractures.

### 3.2. Influence of Fracture-Filling Conditions on Borehole Wave Modes

**Figure 8:**
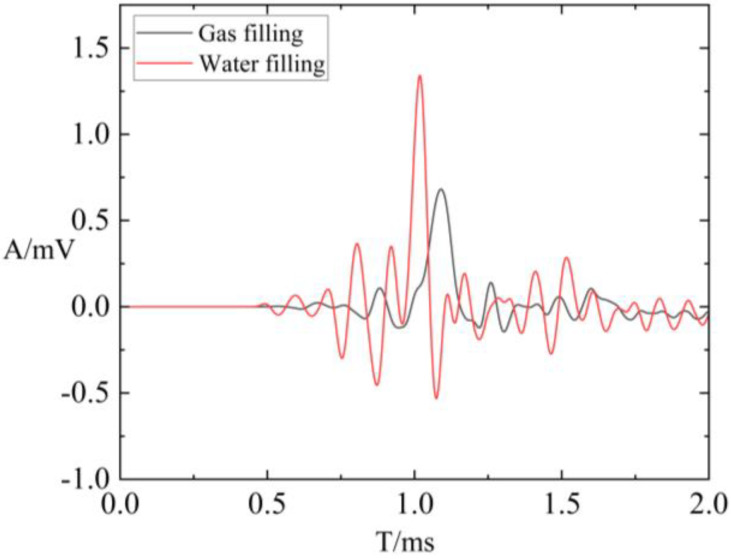
Full waveform under different filling conditions with fracture width of 0.2 mm.

**Figure 9:**
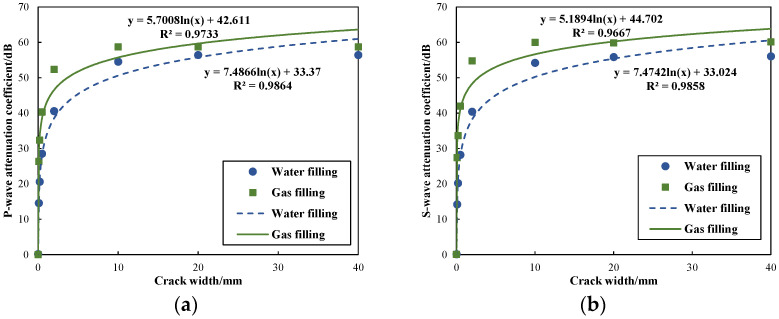
Variation in attenuation coefficients of different fracture filling with fracture widths: (**a**) relation of the attenuation coefficient of P-waves with fracture width; (**b**) relation of the attenuation coefficient of S-waves with fracture width.

To explore the influence of fluid-filling conditions in fractures on borehole wave modes, the filling conditions of fractures in the model were altered. In this scenario, the dominant frequency was set to 8 kHz. The fracture was filled with gas, with a density of 200 kg/m^3^; and an acoustic velocity of 500 m/s, and the fracture width was set to 0.2 mm. Initially, waveform comparisons were conducted for different filling conditions with a fracture width of 0.2 mm, as illustrated in Figure 8. The results show that when fractures are filled with water, the arrival time is minimized, and the amplitude is maximized. Conversely, when fractures are filled with gas, the arrival time is maximized, and the amplitude is minimized. Based on the waveform data presented, attenuation calculations were performed. Figure 9 presents a comparison of fracture width and attenuation coefficients for refracted P-waves and S-waves under varying fracture-filling conditions. It showcases the attenuation coefficients corresponding to fracture widths of 0.1 mm, 0.2 mm, 0.5 mm, 2 mm, 10 mm, 20 mm, and 40 mm. The figure reveals that fractures filled with gas exhibit a significantly larger attenuation rate compared to those filled with water. Furthermore, the attenuation coefficients of P-waves and S-waves are consistently higher for fractures filled with gas compared to those filled with water. This difference is particularly noticeable for S-wave attenuation. It is also observed that when the fracture is filled with gas, the refracted P-waves and S-waves are more sensitive to the change in fracture properties. This heightened sensitivity offers a significant advantage for the identification and evaluation of fractures.

**Figure 10:**
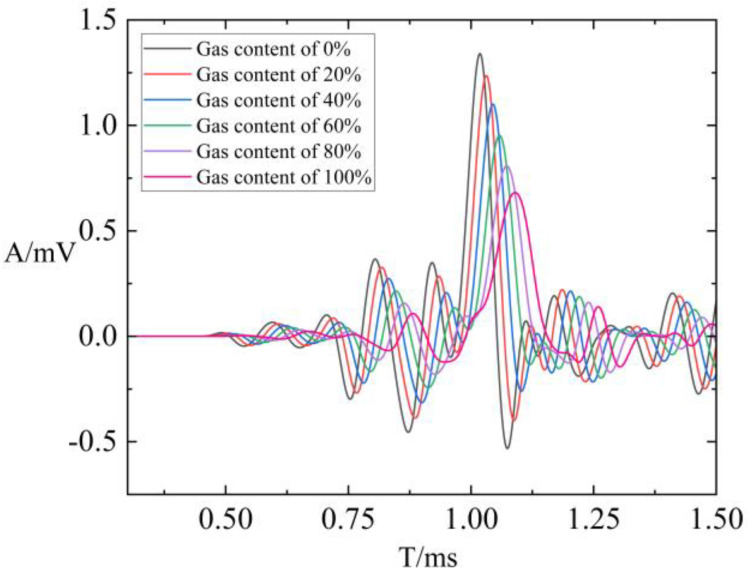
Waveform comparison of different gas saturation with a fracture width of 0.2 mm.

**Figure 11:**
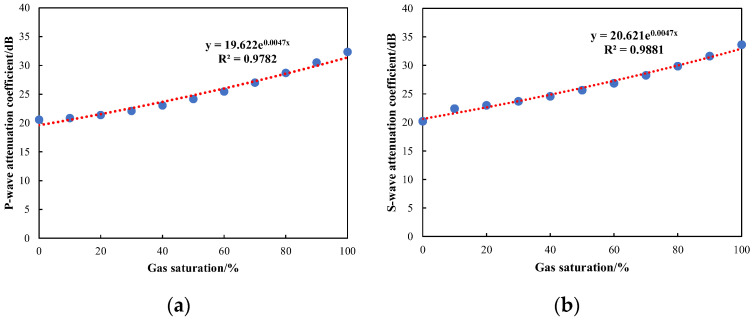
Variation in attenuation coefficients with gas saturation: (**a**) relation of attenuation coefficient of P-waves with gas saturation; (**b**) relation of attenuation coefficient of S-waves with gas saturation.

Subsequently, to further quantify the relationship between gas saturation and attenuation coefficients, the gas saturation in the numerical model was systematically varied from 0% to 100%, with a step size of 10%. The waveforms were analyzed, and the attenuation coefficients of compressional and shear waves were computed. Figure 10 presents a waveform comparison for different gas saturations with a fracture width of 0.2 mm. It highlights that at 0% gas saturation, the arrival time is shortest, and the amplitude is highest. With the continuous increase in gas saturation, the arrival time steadily increased, while the amplitude gradually decreased. Figure 11 illustrates the relationship between compressional and shear wave attenuation coefficients and gas saturation. It is observed that both attenuation coefficients of compressional and shear waves exhibit exponential variations with gas saturation, leading to the derivation of a quantitative relationship. In practical applications of array acoustic logging, the formula can be utilized to achieve quantitative predictions of gas saturation. This offers a novel approach for identifying and predicting fluid properties within fractured reservoirs.

### 3.3. Influence of Acoustic Frequency on Borehole Wave Modes

**Figure 12:**
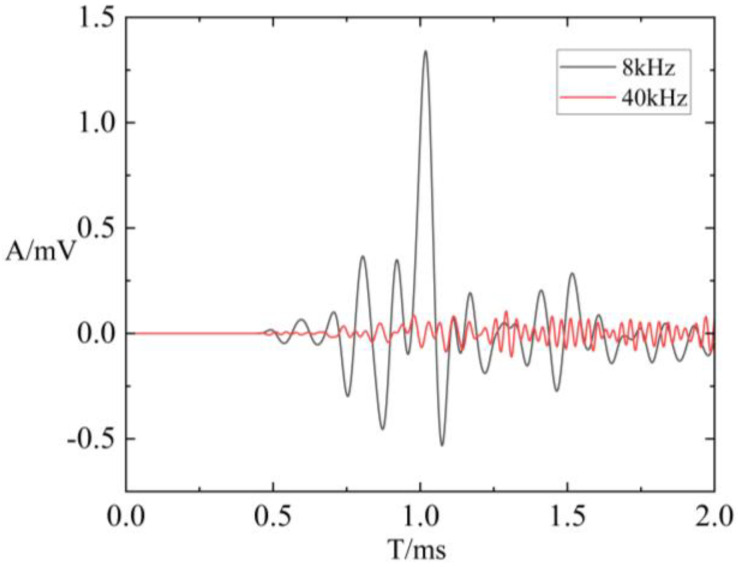
A full-waveform comparison is conducted at different dominant frequencies when the fracture width is 0.2 mm and the Transmitter–Receiver spacing is 1.28 m.

**Figure 13:**
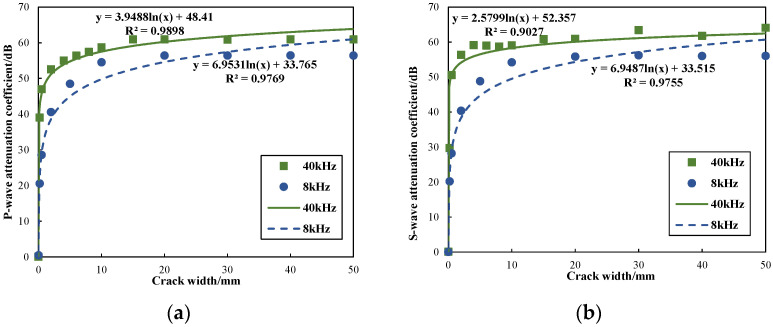
Attenuation coefficient varies with the fracture width at different dominant frequencies: (**a**) relationship between P-wave attenuation coefficient and fracture width; (**b**) relationship between S-wave attenuation coefficient and fracture width.

To explore the impact of source dominant frequency on borehole wave modes, the source dominant frequencies were set to 8 kHz and 40 kHz for numerical calculations. The source dominant frequency in physical experimental research is generally much higher than the dominant frequency of actual well logging. The simulation at 40 kHz aims to study the effect of frequency on energy attenuation in fractures. The results will then be used to guide the evaluation of fractures based on actual array acoustic logging data from physical experiments. Under these two different frequencies, a full-waveform comparison was carried out when the fracture width was 0.2 mm and the Transmitter–Receiver spacing was 1.28 m, as shown in Figure 12. The comparison reveals that, for the same fracture width, the amplitude of the wave modes at the higher frequency (40 kHz) is lower than that at 8 kHz. This phenomenon occurs because the absorption of acoustic signals by the medium increases proportionally to the square of the source dominant frequency. In other words, under the same conditions of fracture width and medium properties, higher-frequency acoustic signals experience faster attenuation. Figure 13 depicts the relationship between refracted compressional and shear wave attenuation coefficients with varying fracture widths at different dominant frequencies. It presents the attenuation coefficients for fracture widths of 0.2 mm, 0.5 mm, 2 mm, 4 mm, 6 mm, 8 mm, 10 mm, 15 mm, 20 mm, 30 mm, 40 mm, and 50 mm. It was evident that when the dominant frequency was 40 kHz, the attenuation coefficients of refracted P-waves and S-waves increased with fracture width at a faster rate compared to when the dominant frequency was 8 kHz. Consequently, under identical fracture width conditions, the refracted compressional and shear wave attenuation coefficients are relatively larger at 40 kHz.

To further quantitatively study the influence of source dominant frequency on the attenuations of borehole wave modes, the attenuation coefficients of borehole wave modes corresponding to the similar fracture width and dominant frequency ratio conditions (the fracture width–frequency product is a constant) were compared and analyzed. The results are presented in Table 2. As shown in Table 2, for a specific fracture width (e.g., 2 mm) at 40 kHz, the wave modes’ attenuation coefficient is similar to the coefficient for a five-fold-wider fracture (e.g., 10 mm) at 8 kHz. The ratio of attenuation coefficients of wave modes at a fracture width ratio similar to the dominant frequency ratio (the product of fracture width and dominant frequency is a constant) was further calculated, as shown in Table 3. It can be observed that, when the product of fracture width and dominant frequency remains constant, the corresponding attenuation coefficient ratios approach 1. This indicates that the attenuation coefficients of wave modes corresponding to fracture widths at different frequencies are the same. In order words, the attenuation process of acoustic propagation in fractured media follows the principle of acoustic similarity. In the actual application of array acoustic logging fracture evaluation, the research results can be utilized to correct the frequency effects, thereby enhancing the accuracy of fracture attribute parameter assessment.

## 4. Conclusions

This paper utilized a three-dimensional variable grid finite difference program to simulate array acoustic logging at different frequencies. The simulations were based on a numerical model of a fractured borehole formation with varying fracture widths and distinct fluid-filling conditions. By processing the waveform data, we extracted velocity and amplitude attenuation characteristics of refracted P-waves and S-waves. Subsequently, we analyzed the extracted characteristic parameters of refracted compressional and shear waves for the fracture properties. Furthermore, an analysis was conducted to deduce the quantitative variations in the characteristic parameters of refracted compressional and shear waves. The main conclusions of this study are as follows:

(1) The attenuation coefficients of refracted P-waves and S-waves exhibit a logarithmic increase as the fracture width increases. Particularly, when the fracture width was within 2 mm, there was a significant rise in the attenuation coefficients of refracted P-waves and S-waves, indicating their sensitivity to micro-fractures. However, as the fracture width expanded to 10 mm, their attenuation coefficients stabilized, exhibiting a decreasing rate of attenuation increase. This suggests that P-waves and S-waves exhibit lower sensitivity to macro-fractures.
(2) When the source dominant frequency and fracture width are fixed, fractures filled with gas experience faster attenuation compared to those filled with water. The attenuation coefficients of compressional and shear waves in gas-filled fractures consistently exceed those in water-filled fractures, with the difference being particularly noticeable in shear wave attenuation. Furthermore, through quantitative analysis, it was observed that the attenuation coefficients of both refracted compressional and shear waves exhibit an exponential variation with gas saturation. Under the determined fracture parameters, this functional relationship can be further utilized for the quantitative prediction of gas saturation in fractures. It provides a new means for identifying and predicting fluid properties in fractured reservoirs.
(3) When the fracture width and filling conditions were fixed, the amplitudes of the compressional and shear waves were smaller when the dominant frequency was 40 kHz than when the dominant frequency was 8 kHz. Additionally, the attenuation of wave modes at 40 kHz was faster than that at 8 kHz. Further quantitative analysis revealed that, under different frequency conditions, the attenuation coefficients of wave modes corresponding to fractures with different widths exhibit a consistent pattern. At different frequencies, the ratios of wave modes’ attenuation coefficients for similarly scaled fracture widths (constant fracture width–frequency product) closely approach 1. This indicates that the acoustic propagation attenuation process in fractured media follows the principle of acoustical similarity. The influence of frequency on attenuation demonstrates a consistent proportionality. This conclusion provides a reference for frequency calibration in actual fracture evaluation based on acoustic logging, thereby enhancing the accuracy of fracture evaluation.

In summary, this study reveals the effects of fracture width, filling condition, and acoustic frequency on borehole wave modes, providing a theoretical basis for developing novel methods to evaluate and correct fracture attribute parameters based on array acoustic logging information.

## Figures and Tables

**Table 1:** Media parameters of the model.

	$Vp/(m\cdot s^{-1})$	$Vs/(m\cdot s^{-1})$	$\rho /(kg\cdot m^{-3})$
Formation	4000	2300	2300
Borehole and fracture fluids	1500	—	1000

**Table 2:** Statistical table of P-wave and S-wave attenuation coefficients for fracture widths with similar ratios at different frequencies.

Source dominant frequency/kHz	40					8				
Width of fracture/mm	2	4	6	8	10	10	20	30	40	50
P-wave attenuation coefficient/dB	52.566	54.999	56.375	57.435	58.696	54.512	56.410	56.410	56.433	56.410
S-wave attenuation coefficient/dB	56.344	59.107	58.993	58.727	59.086	54.215	55.861	56.241	56.039	56.077

**Table 3:** Ratio of P-wave attenuation and S-wave coefficients of similar ratio fracture widths (constant fracture width–frequency product) at different frequencies.

Source dominant ratio	40:8				
Fracture width ratio	2:10	4:20	6:30	8:40	10:50
P-wave attenuation coefficient ratio of similar ratio to fracture width at different frequencies	0.964	0.975	0.999	1.018	1.041
S-wave attenuation coefficient ratio of similar ratio to fracture width at different frequencies	1.039	1.058	1.048	1.048	1.053

## Data Availability

Citing the confidentiality of laboratory data, this paper refrains from the public disclosure of the experimental results. Data sharing is not applicable to this article.
